# Regulating digital health

**DOI:** 10.2471/BLT.20.020420

**Published:** 2020-04-01

## Abstract

Realizing the potential of digital health requires overcoming its inherent risks. Gary Humphreys reports.

On a January night in 2019, somewhere in the United States of America (USA), Yisroel Mirsky waited until the cleaning staff opened the doors, then entered the hospital premises and installed his device in a computerized tomography (CT) scanner room.

Hidden under a panel in the floor, the device accessed the hospital’s picture archiving and communication system (PACS).

PACS are a fixture of many hospitals and exemplify both the advantages and disadvantages of digitization. One of those disadvantages is vulnerability to intrusion.

“PACS are designed to be accessible and many are connected to the web for remote access, enabling search engines, such as Shodan.io to find them,” Mirsky explains, “and because they generally have to work with older equipment that cannot support encryption, often the internal traffic is unencrypted or misconfigured.”

Using a piece of artificial intelligence (AI) software known as a generative adversarial network, the device started altering CT scans of patients’ lungs, inserting or removing tumours, as appropriate.

“We did it to reveal vulnerabilities in the PACS,” says Mirsky, who is a researcher at the Ben-Gurion University Cyber Security Research Center in Be’er Sheva, Israel, adding that it was done with the hospital’s full consent and that the patients were not affected by the experiment.

That a hospital’s digital systems should be so open to cyber-attack and that AI should be the weapon used does not surprise Timo Minssen, Managing Director of the Centre for Advanced Studies in Biomedical Innovation Law in Copenhagen.

“Web-connected digital health systems are highly vulnerable to attack,” he says, “and like any other technology, AI can be used for both beneficial and harmful ends.”

Generally, hackers have no need for sophisticated AI, relying on simpler “ransomware” that encrypts hospital data. The 2017 WannaCry ransomware attack is one high-profile example. The attack shut down work at 16 hospitals across the United Kingdom of Great Britain and Northern Ireland by blocking access to patient records and other services for weeks. There have been multiple attacks since then.

Minssen has been studying digital health systems for three years to help develop legislation that promotes the benefits and mitigates the risks.

“The risks reflect the broad array of socioeconomic, ethical and legal issues raised by the applications of digital technologies generally and AI in particular, ranging from patient privacy to transparency and accountability,” he says.

“Machine learning is a major concern for medical authorities.”

Timo Minssen

Privacy is a major concern especially with regard to medical information that may have an impact on a person’s insurability or employability. “Recent examples of data misuse by Facebook and others highlight the importance of reconciling technological possibilities with the protection of privacy,” Minssen says, “but even where information is being collected for legitimate purposes – such as for epidemiological or clinical research – privacy is an issue.”

To date regulators have sought to address this issue by introducing patient consent and anonymization clauses into their statutes. For example, Europe’s General Data Protection Regulation (GDPR), which governs the use of personal data, including health, biometric and genetic data, requires that consent be obtained from patients regarding the use of their data. It also includes research exemptions, imposing anonymization where consent is not obtained. In February 2020, the European Commission published an AI strategy that stressed continued enforcement of the GDPR to protect personal data and privacy.

Anonymisation is supposed to make it impossible for data to be linked to an individual, but some researchers have questioned anonymisation’s effectiveness. An article published in the July 2019 issue of *Nature Communications, *for example,**revealed that anonymised datasets could be traced back to individuals using machine learning.

The risk of privacy breaches increases where regulation is absent or poorly enforced. In India, a digitized social welfare system known as Aadhaar has come under scrutiny in this regard, with multiple privacy breaches undermining trust in the system. A personal data bill is being reviewed by a joint parliamentary committee. If passed, the bill will establish a national Data Protection Authority.

Pam Dixon, Executive Director of the World Privacy Forum, a research group that focuses on data privacy, assessed the Aadhaar system between 2010 and 2016, and questions whether the tightening of regulation in India will make much difference.

“Aadhaar is a good example of a biometrically-based digital programme reaching a point of pervasiveness, which makes it very difficult to regulate,” she says.

Dixon believes that it is the pervasiveness of the digital ecosystem that makes it such a cause for concern.

She worries about the accumulation and sharing of individual data, which are then used to establish predictive health scores, which are widely disseminated.

In the USA, examples include the Affordable Care Act health risk score, which creates a relative measure of predicted health care costs for a given enrollee and can serve as a proxy score for how sick a person is. Another is the brand name medicine propensity score, which is used to predict how likely you are to choose brand-name medications over generics.

 “These scores are traded by data brokers and used by corporate clients that include pharmaceutical companies and insurers,” she says. “Increasingly they are being used to feed the algorithms on which AI is based and predictions made. We need to be very careful to ensure that the decisions and predictions AI is making do not cross ethical lines.”

The OECD published a set of principles calling for appropriate safeguards, such as allowing for human beings to understand AI-based outcomes and challenge them. The European Commission strategy of February 2020 calls for AI systems that are transparent and traceable, and that allow for human oversight.

“The fact that we are now discussing the need to understand AI-based outcomes and challenge them is a reflection of the autonomy AI is beginning to achieve with neural networks and machine learning,” says Minssen.

Minssen argues that this autonomy and the so-called black box algorithms on which machine learning is based, are presenting regulators with unprecedented challenges.

“We need to […] ensure that the decisions and predictions AI is making do not cross ethical lines.”

Pam Dixon

“Machine learning is a major concern for medical authorities, such as the European Medicines Agency or the US Food and Drug Administration,” he says. “They are intensively searching for experts who have experience in artificial intelligence and machine learning, and who also have an understanding of how this technology will evolve.”

Professor Leong Tze Yun, Director of AI Technology at AI Singapore, a national program on Artificial Intelligence, acknowledges the concern about so-called black box algorithms, but argues that such algorithms can still be used to build a transparent and trusted AI system as long as the assumptions and limitations, operational protocols, data properties, and output decisions can be systematically examined and validated.

“AI systems can be “transparent”, without the need for every computational step to be traceable,” she says.

Dixon takes a similar view. “You don’t have to understand automatic transmissions in order to regulate the kinds of cars we allow on the streets,” she says. “You just have to set appropriate performance and safety parameters and establish clear rules for drivers.”

Minssen is not convinced that regulating AI in health is going to be so simple. “Complexity and transparency issues aside, what do we do when the algorithm whose recommendations a doctor is following is the cause of harm to a patient? Is the doctor liable? Or is it the algorithm?”

What about people who want to cause harm such as the hackers who shut down a cancer centre in Oahu, Hawaii, USA, in December 2019, disrupting the delivery of radiotherapy there?

For Yisroel Mirsky, pushing back against the hackers must begin with hospitals taking the issue of cyber-security seriously. Along with a group of colleagues, Mirsky published a study based on the penetration test he carried out in the USA in 2019 in the *Proceedings of the 28th USENIX Security Symposium* in June 2019. He was surprised by the response among the hospital administrators who read it.

“People recognized the problem, and some tightened up their security with encryption,” he says. “but most said that the investment required to address their legacy issues and make their systems secure was too great for them to envisage.”

The cost of not making the investment required in cyber security may be far greater, as will the cost of not addressing the serious ethical and regulatory issues raised by digital health.

According to Bernardo Mariano, director of the World Health Organization’s Department of Digital Health and Innovation, digital health security, regulations and ethics will be among the issues discussed when WHO’s global strategy on digital health is presented to member states at the World Health Assembly in May 2020.

“In addition to policies, the global community needs to collectively establish a health data intelligence monitoring system or framework, to detect and report on health data misuse and all associated cyber security risks,” he says.

**Figure:**
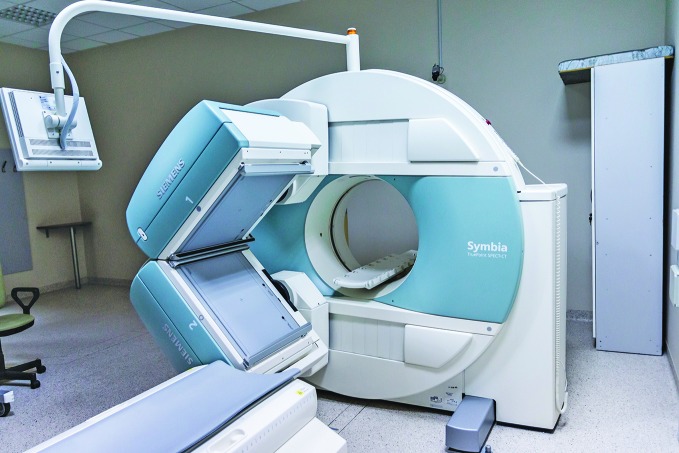
Digital imaging devices generate sensitive data that require secure storage

PIXABAY/Jarmoluk

**Figure:**
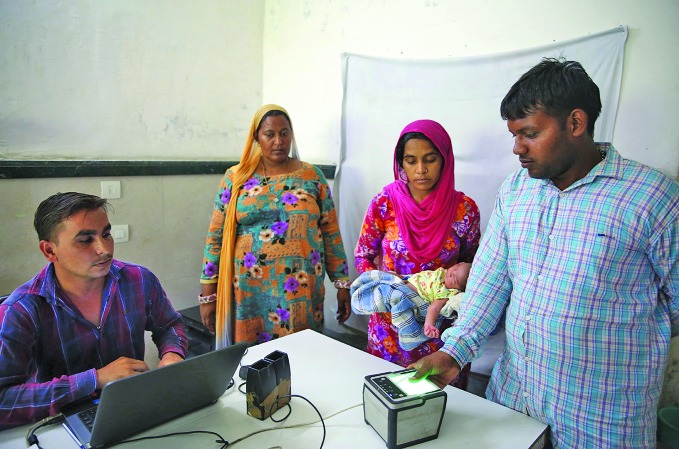
A man enters biometric data to register his newborn in India’s Aadhaar system

UNICEF/Kapoor

